# Encapsulation Efficiency and Functional Stability of Cinnamon Essential Oil in Modified *β*-cyclodextrins: In Vitro and In Silico Evidence

**DOI:** 10.3390/foods12010045

**Published:** 2022-12-22

**Authors:** Kegang Wu, Tong Zhang, Xianghua Chai, Xuejuan Duan, Dong He, Hongpeng Yu, Xiaoli Liu, Zhihua Tao

**Affiliations:** School of Chemical Engineering and Light Industry, Guangdong University of Technology, Guangzhou 511443, China

**Keywords:** cinnamon essential oil, encapsulation, cyclodextrins, molecular docking, antibacterial, antioxidant

## Abstract

Essential oils (EOs) have good natural antioxidant and antimicrobial properties; however, their volatility, intense aroma, poor aqueous solubility, and chemical instability limit their applications in the food industry. The encapsulation of EOs in *β*-cyclodextrins (*β*-CDs) is a widely accepted strategy for enhancing EO applications. The complexation of cinnamon essential oil (CEO) with five types of *β*-CDs, containing different substituent groups (*β*-CD with primary hydroxyl, Mal-*β*-CD with maltosyl, CM-*β*-CD with carboxymethyl, HP-*β*-CD with hydroxypropyl, and DM-*β*-CD with methyl), inclusion process behaviors, volatile components, and antioxidant and antibacterial activities of the solid complexes were studied. The CEOs complexed with Mal-*β*-CD, CM-*β*-CD, and *β*-CD were less soluble than those complexed with DM-*β*-CD and HP-*β*-CD. Molecular docking confirmed the insertion of the cinnamaldehyde benzene ring into various *β*-CD cavities via hydrophobic interactions and hydrogen bonds. GC-MS analysis revealed that HP-*β*-CD had the greatest adaptability to cinnamaldehyde. The CEO encapsulated in *β*-, Mal-*β*-, and CM-*β*-CD showed lower solubility but better control-release characteristics than those encapsulated in DM- and HP-*β*-CD, thereby increasing their antioxidant and antibacterial activities. This study demonstrated that *β*-, Mal-*β*-, and CM-*β*-CD were suitable alternatives for the encapsulation of CEO to preserve its antioxidant and antibacterial activities for long-time use.

## 1. Introduction

Cinnamon, a traditional herbal medicine, belonging to the *Lauraceae* family, which is widely distributed in China, Sri Lanka, Vietnam, Madagascar, India, and the Seychelles [1]. Essential oils (EOs), which are naturally derived perfumed liquids, are the major components of cinnamon. They possess broad-spectrum antimicrobial activity and excellent antioxidant ability [2]. EOs increase bacterial cell membrane permeability, which causes the leakage of ions and cytoplasmic contents, thus inhibiting the physiological activity of the bacterial cell [3]. However, CEO phytochemicals are chemically unstable, particularly cinnamaldehyde oxidative susceptibility. Moreover, the volatility, low water solubility, and intense aroma greatly limit their applications in the food industry [4].

**Figure 1:**
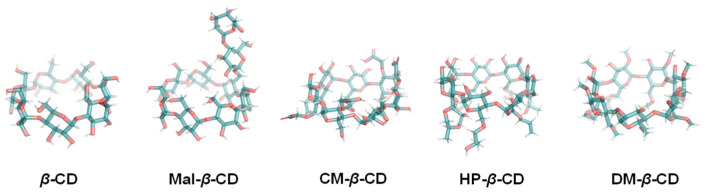
Structures of *β*-CD, Mal-*β*-CD, CM-*β*-CD, HP-*β*-CD, and DM-*β*-CD.

Encapsulation is one of the most widely used techniques to overcome these limitations. Shell materials that can be used for the encapsulation of EOs include gums, chitosan, maltodextrins, starch, and proteins [5]. Among these shell materials, cyclodextrins (CDs) have been extensively used to encapsulate EOs. CDs are cyclic oligomers consisting of *a*-1, 4-linked D-glucopyranose units. CDs are widely used to protect flavor substances, vitamins, and natural pigments, thereby improving the physical and chemical properties of these substances. The secondary structure of a CD molecule is formed via H-bonds, which endows *β*-CDs with a rather rigid structure and, thus, low water solubility but good stability [6]. However, the low aqueous solubility and the nephrotoxicity of *β*-CDs restrict their applications [7]. The structural alterations of *β*-CDs including maltosyl-*β*-cyclodextrin (Mal-*β*-CD), O-carboxymethyl-*β*-cyclodextrin (CM-*β*-CD), (2,6-di-o-methyl)-*β*-cyclodextrin (DM-*β*-CD), and hydroxypropyl-*β*-cyclodextrin (HP-*β*-CD) (Figure 1) have been developed to overcome these limitations and meet market requirements (especially in the food industry).

Encapsulation in modified *β*-CDs affects the stability, solubility, and bioactive properties of different compounds, such as *piper nigrum* EO encapsulated in Mal-*β*-CD, thymol EOs [8] encapsulated in *β*-CD, rutin [9] encapsulated in CM-*β*-CD, citrus aurantium L. EO [10] encapsulated in HP-*β*-CD, and quercitrin [11] encapsulated in DM-*β*-CD. The tested cyclodextrins increased the solubility of these EOs and the stability of the inclusion complexes played a crucial role in their control-release characteristics as well as their biological activity. However, the effect of CDs on the biological activity of EOs remains unclear.

The use of *β*-CD and HP-*β*-CD for EOs has been described, whereas there are no reports on EO encapsulation using Mal-*β*-CD, CM-*β*-CD, or DM-*β*-CD. Kfoury et al. [12] compared the inclusion behavior of six EOs encapsulated in four types of CDs, including HP-*β*-CD. Samperio et al. [13] investigated the solubility of 23 plant EOs and 4 parabens encapsulated in α- and *β*-CD. Exploring more sources of CDs for EO encapsulation to obtain higher stability and better control-release characteristic is of great interest for modulating the biological activity of EOs.

In this study, we investigate the effects of aqueous solubility and stability of CD-cinnamon essential oil (CEO) complexes on their antioxidant ability and antibacterial activity against food-borne bacteria and explore the potential of using new sources of *β*-CDs (Mal-*β*-CD, CM-*β*-CD, and DM-*β*-CD) in contrast to the commonly used *β*-CDs (*β*-CD and HP-*β*-CD) for CEO encapsulation with enhanced properties. Furthermore, we determine the differences in the volatile constituents of CEOs encapsulated in various CDs via gas chromatography-mass spectrometry (GC-MS) and examine the interaction force of inclusion via molecular docking to assess the relationship between volatile compounds and their antioxidant/antibacterial activity.

## 2. Materials and Methods

### 2.1. Material and Chemicals

*Escherichia coli* (ATCC8739), *Staphylococcus aureus* (ATCC65389), *Salmonella choleraesuis* (GM 11163), and *Pseudomonas aeruginosa* (GM 11220) were purchased from the Guangdong Institute of Microbiology (Guangzhou, China).

Cinnamon essential oil (CEO), beta-cyclodextrin (*β*-CD), hydroxypropyl-beta-cyclodextrin (HP-*β*-CD), maltosyl-beta-cyclodextrin (Mal-*β*-CD), O-carboxymethyl-beta-cyclodextrin (CM-*β*-CD), and (2,6-di-o-methyl)-β-cyclodextrin (DM-*β*-CD) were supplied by Xiangsixinqing Health Technology Co., Ltd. (Guangzhou, China). The nutrient agar was purchased from Guangdong Huankai Microbial Sci. & Tech. Co., Ltd. (Guangzhou, China). 2-Phenyl-4,4,5,5-tetramethylimidazoline-1-oxyl 3-oxide (PTIO) (CAS 18390-00-6) and the 1,1-diphenyl-2-picryl-hydrazyl radical (DPPH) (CAS 1898-66-4) was obtained from Biohonor Technology Co., Ltd. (Guangzhou, China). The total antioxidant capacity assay kit was obtained from Suzhou Grace Biotechnology Co., Ltd. (Suzhou, China). Other unmentioned reagents used in this work were of analytical grade. Deionized water was used to perform all the experiments.

### 2.2. Phase Solubility

The complexation of CEO in modified *β*-CDs was performed using phase solubility diagrams according to Higuchi and Connors et al. [14] with some modifications. The excess CEO was added to 25 mL of aqueous solutions containing CDs (Mal-, CM-, HP-, DM-, and *β*-CD) at different concentrations (0–50 mM) under continuous stirring for 24 h at 20 °C in the dark. After 24 h of reaction, the solutions were filtered using 0.45 μm cellulose acetate membrane filters for UV-Vis (TU-1950, Beijing Purkinje General Instrument Company, Beijing, China) assays. Cinnamaldehyde was used as the guest molecule to determine the inclusion behavior.

The complexation constant (Kc) between cinnamaldehyde and each type of *β*-CD was calculated from both the slope of the phase solubility diagram and the solubility of the compound aqueous solution (S_0_) by using Equation (1):

(1) $$K_{c}\left(L/\mathrm{mol}\right)=\frac{\mathrm{slope}}{S_{0}\times \left(1-\mathrm{slope}\right)}$$

### 2.3. Preparation of Modified β-cyclodextrin Cinnamon Essential Oil Inclusion Complexes

The *β*-CDs solution was prepared by adjusting the water: *β*-CDs ratio to 10:1. Then, a CEO-ethanol solution was then injected into the *β*-CDs solution and stirred at 300 rpm in the dark. The reaction parameters for the ratio of CEO to shell materials, time, and temperature are listed in Table 1. The mixture was stirred at 300 rpm for 6–8 h at 25 °C and the solutions containing the complexes were freeze-dried. The reaction parameters were determined using single-factor and orthogonal experiments (See Supplementary Materials).

The CEO solutions were freeze-dried in a freeze dryer (Scientz-18ND, Ningbo Xinzhi Bioscience Co., Ltd., China) at −54 °C for 12 h after freezing at −80 °C for 24 h. The freeze-dried solid complexes obtained were stored in a drying dish until used.

The CEO content was determined using a UV-Vis spectrophotometer (TU-1950, Beijing Purkinje General Instrument Company, Beijing, China) according to a previously described method with some modifications [15]. Different concentrations of CEO standard solutions (1, 2, 3, 4, 5, and 6 μL/L) were prepared using absolute ethanol and the absorbance of the solutions was measured at 284 nm with ethanol absolute as the blank control. 

The dehydration yield (DY) was calculated using Equation (2):

(2) $$\mathrm{DY}\;\left(\%\right)=\frac{\mathrm{solid}\;\mathrm{complexes}\;\mathrm{obtained}\;\left(g\right)}{\mathrm{total}\;\mathrm{solid}\;\mathrm{added}\;\mathrm{in}\;\mathrm{solution}\;\left(g\right)}\times 100\%$$

The encapsulation efficiency (EE) was calculated using Equation (3):

(3) $$E\;\left(\%\right)=\frac{\mathrm{total}\;\mathrm{CEO}\;\mathrm{encapsulated}\;\mathrm{in}\;\mathrm{solid}\;\mathrm{complex}\;\left(\mathrm{mg}\right)}{\mathrm{initial}\;\mathrm{total}\;\mathrm{CEO}\;\mathrm{added}\;\mathrm{in}\;\mathrm{solution}\;\left(\mathrm{mg}\right)}\times 100\%$$

The CEO load (DL) was calculated using Equation (4):

(4) $$\mathrm{DL}\;\left(\frac{\mathrm{mg}}{g}\right)=\frac{\mathrm{total}\;\mathrm{CEO}\;\mathrm{encapsulated}\;\mathrm{in}\;\mathrm{solid}\;\mathrm{complex}\;\left(\mathrm{mg}\right)}{\mathrm{total}\;\mathrm{solid}\;\mathrm{complexes}\;\left(g\right)}\times 100\%$$

### 2.4. Scanning Electron Microscopy (SEM)

The samples were sputtered with gold under reduced pressure and analyzed using SEM (SU8220; Tokyo, Japan) at an accelerating voltage of 10 kV and various magnifications [16].

### 2.5. Molecular Docking

Gaussian 09 software and AutoDockTools 1.5.7 software were used to predict the most likely optimal configurations of various inclusion complexes. Based on the crystallographic data of native *β*-CD and DM-*β*-CD available in the Cambridge Structural Database, the binding modes of Mal-*β*-CD, CM-*β*-CD, and HP-*β*-CD were developed using Gaussian 09 by adding the corresponding groups to *β*-cyclodextrin and further subjected to complete energy minimization using density functional theory (DFT). The molecular structure of cinnamaldehyde was obtained from the Cambridge Crystallographic Data Centre (CCDC). Cinnamaldehyde was used as the guest molecule to determine the inclusion behavior. The docking between cinnamaldehyde and various modified *β*-CD was then carried out in AutoDock by the insertion of cinnamaldehyde into various *β*-CD cavities according to the best host-guest relative orientation [17,18].

### 2.6. GC-MS Analysis

The volatile compounds in the inclusion complexes were extracted using the method described by Muhoza et al. [19]. The dried particles of *β*-CD-ICs, Mal-ICs, CM-ICs, HP-ICs, and DM-ICs were added to a beaker containing n-hexane. Ultrasound sonication for 90 min at 25 °C was used to extract CEOs. The CEOs were collected via centrifugation at 2000 rpm for 10 min and concentrated using a rotary evaporator (RE-52 A, Yarong Co. Ltd., Shanghai, China).

The samples were analyzed using a 7890 B gas chromatograph (Agilent Technologies, Santa Clara, CA, USA) equipped with a 5977 B mass spectrometer (Agilent). The analytes were separated in HP-5 ms columns (60 m × 0.25 mm × 0.25 μm). The temperature program of the column oven was set as follows: the injector temperature was 250 °C and the initial temperature was 50 °C, which was maintained for 3 min and then gradually increased to 180 °C at a rate of 2 °C/min. The temperature was further increased to 300 °C at a rate of 20 °C/min and maintained for 10 min. The linear velocity of the carrier gas was 1.0 mL/min at a split ratio of 50:1. The mass spectrometry detector had an ion source temperature of 230 °C and a mass scan range of 29–400 amu.

The samples were analyzed with using an Agilent 5977 B and the spectrum peak was determined using the standard NIST14 standard mass spectrometry library. The components were confirmed with reference to the standard map and the related literature and the relative content of each component was calculated using the peak area normalization, which means the relative content of each component was calculated using Equation (5).

(5) $$\mathrm{Relative}\;\mathrm{content}\;\left(\%\right)=\frac{\mathrm{the}\;\mathrm{peak}\;\mathrm{area}\;\mathrm{of}\;\mathrm{each}\;\mathrm{component}}{\mathrm{the}\;\mathrm{sum}\;\mathrm{of}\;\mathrm{all}\;\mathrm{the}\;\mathrm{peak}\;\mathrm{area}}\times 100\%$$

### 2.7. Antioxidative Activities

#### 2.7.1. Determination of Ferric Reducing Antioxidant Power (FRAP Assay)

The FRAP assay was performed as previously described [20,21] with slight modifications. Briefly, the freshly prepared FRAP reagent was heated to 37 °C. After adding 100 μL CEO samples (or CD-CEO complexes equivalents to 100 uL CEO) and 100 μL ultrapure water to 850 μL FRAP reagent, the mixture was incubated at 25 °C for 10 min; 200 μL ultrapure mixed with 850 μL FRAP reagent used as a control. The absorbance values of the solutions were determined at 590 nm using a UV-Vis spectrophotometer. The reducing power of the samples was estimated using a standard calibration curve and the results are presented as the Trolox equivalent antioxidant capacity (μmol Trolox/100 g).

#### 2.7.2. DPPH Radical Scavenging Activity

The DPPH radical scavenging activity of the samples was measured using a previously reported method, with some modifications [22]. Ten microliters of CEO (or CD-CEO complexes equivalent to 10 μL CEO) was prepared with 1490 μL ethanol. A 0.1 mM DPPH solution was prepared with ethanol. Subsequently, 1500 μL of the CEO solution was mixed with 1500 μL of the DPPH solution. Deionized water was used as the control instead of the oil solution and ethanol was used as the blank. The mixture was agitated using a vortex and kept in the dark at 25 °C for different times (4, 12, 24, 36, 48, 60, and 72 h), following which the absorbance was measured at 517 nm using a UV-Vis spectrophotometer. The DPPH radical scavenging rate was calculated using Equation (6):

(6) $$\mathrm{Scavenging}\;\mathrm{rate}\;\left(\%\right)=\left(1-\frac{As-Ac}{A}\right)\times 100\%$$

where, *A*s is the absorbance of the sample reaction solution, *A*c is the absorbance of the solution containing 3 mL of ethyl alcohol and samples, and *A* is the absorbance of the solution containing 1.5 mL of DPPH and 1.5 mL of ethyl alcohol.

#### 2.7.3. PTIO Radical Scavenging Activity

The PTIO radical scavenging ability was determined as previously described [23]. Briefly, 10 μL CEO (or CD-CEO complexes equivalents to 10 μL CEO) was prepared with 1 mL deionized water. The PTIO solution (1.7 mM, 2 mL) was mixed thoroughly with 1 mL EOs solution and incubated in the dark at 25 °C for different times (6, 24, 48, 72, 96, 120, and 144 h). The absorbance at 557 nm was measured using a UV-Vis spectrophotometer. The PTIO radical scavenging rate was calculated using Equation (7):

(7) $$\mathrm{Scavenging}\;\mathrm{rate}\;\left(\%\right)=\left(1-\frac{A_{m}-A_{n}}{A}\right)\times 100\%$$

where *A*m is the absorbance of the sample reaction solution, *A*n is the absorbance of the solution containing 3 mL of deionized water and samples, and *A* is the absorbance of the solution containing 2 mL of PTIO and 1 mL of deionized water.

### 2.8. Antimicrobial Assay

#### 2.8.1. Preparation of Four Kinds of Pathogens

The strains were incubated on LB agar plates at 37 °C for 24 h. The corresponding single colonies were picked and a 0.5 unit suspension was prepared via Mew’s turbidimetry (1.5 × 10^8^ CFU/mL). The bacterial cultures were then diluted to approximately 10^5^–10^6^ CFU/mL with sterile water.

#### 2.8.2. Determination of Minimum Inhibitory Concentration (MIC)

The MIC value was determined as previously reported [1]. First, the CEO and modified-*β*-CD-ICs were serially diluted in a nutrition broth, with concentrations ranging from 0.05% to 1.6% (*w*/*w*). The diluted antimicrobial solution (1 mL) was mixed with plate count agar (PCA; 14 mL) and poured into plates. Furthermore, 100 μL (10^5–^10^6^ CFU/mL) of pathogenic bacteria was evenly inoculated onto the plates and sterile saline was used as a control. After inoculation, the plates were incubated at 37 °C for 1–10 days to observe differences in strain growth. The lowest concentration of CEO and modified-*β*-CD-ICs without strain growth after incubation at different times was considered for the MIC.

### 2.9. Statistical Analysis

Each assay was performed in triplicate and the data were expressed as means ± standard deviations. The statistical analyses were performed using SPSS software (version 21.0; IBM, Armonk, NY, USA) and differences between the groups were examined using one-way ANOVA with a Waller-Duncan’s multiple range test (*p*-value < 0.05).

## 3. Results and Discussion

### 3.1. Phase Solubility

**Figure 2:**
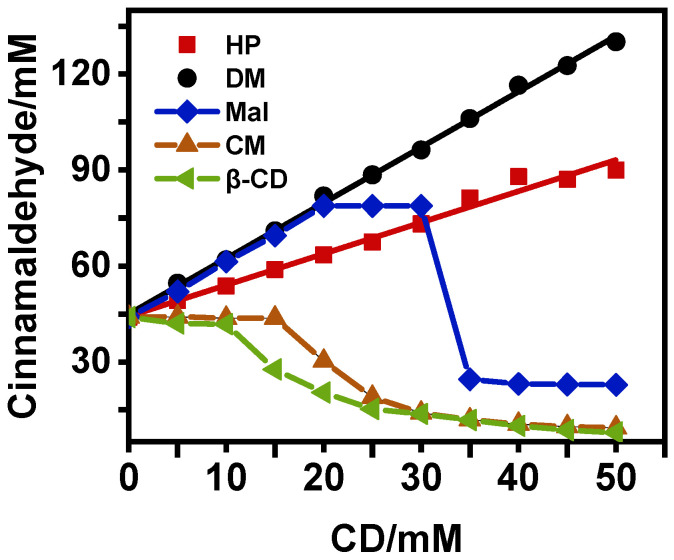
Phase solubility diagrams of CEO with DM- (●), HP- (■), Mal- (◆), CM- *β*-CD (▲), and *β*-CD (◀) in aqueous solution.

The phase solubility curves of cinnamaldehyde with various modified-*β*-CD are presented in Figure 2.

The curves of DM- and HP-*β*-CD showed a linear relationship that is considered A-type according to Loftsson’s study [24], indicating a complexation reaction in which cinnamaldehyde solubility increases as the *β*-CDs concentration increases. In the A-type, the inclusion complexes dissolve easily. The complexation constant (K_c_) of HP-*β*-CD was 1059.5 mol/L. The slope of the solubility diagrams for HP-*β*-CD was lower than 1, indicating that the stoichiometry (HP-*β*-CD: cinnamaldehyde) of the complex was 1:1, whereby each molecule of cinnamaldehyde entered one molecule of CD, which could be considered an A_L_ type profile. This can be explained as follows: Loftsson [24] pointed out that when the drug is complexed with CDs at a ratio of n:1, the inclusion isotherm is linear (A_L_ type). When n is 1, the slope of the line can be calculated using Equation (8) and it is thus clear that the slope is lower than 1:

(8) $$\mathrm{slope}=\frac{S_{0}K}{S_{0}K+1}$$

where S_0_ is the solubility of the compound in the aqueous solution and K is the complexation constant.

However, the slope of DM-*β*-CD was higher than 1. Loftsson [24] estimated that when n is greater than or equal to two, the slope of the A_L_ type line is lower than n. Moreover, a solution exists with cyclodextrin inclusion system and surfactant micelle solubilization system, formed from the aggregation of cyclodextrin or its wrapped inclusion molecule, can also result in a slope greater than 1. It can be predicted that the ratio of complexing for cinnamaldehyde and DM-*β*-CD was greater than 1:1. Moreover, the inclusion complexes with an inclusion ratio of 1:1 further solubilize the cinnamaldehyde molecules in a micelle mode to form a non-inclusion complex, which can also result in a slope greater than 1 [25].

In contrast, the phase solubility curves of *β*-CD, Mal-*β*-CD, and CM-*β*-CD showed a nonlinear relationship, which can be interpreted as low solubility inclusion complexes (B type) [26]. For Mal-*β*-CD, the solubility of cinnamaldehyde increased when the concentration of Mal-*β*-CD increased from 0 to 20 mM and then the platform reached a concentration range of 20–30 mM, whereas the inclusion complexes gradually precipitated. When all the guest molecules were converted into inclusion complexes, the solubility of the inclusion complexes decreased as the concentration of cyclodextrins ranged from 30 to 50 mM. The phase solubility behavior of Mal-*β*-CD was consistent with that of the B_S_ type. Moreover, *β*-CD and CM-*β*-CD were considered B_I_ types when complexed with cinnamaldehyde. As shown in Figure 2, the solubility of cinnamaldehyde was constant for *β*-CD and CM-*β*-CD as the concentration increased from 0 to 10 mM, indicating that *β*-CD-ICs and CM-ICs are insoluble in water. At concentrations of 10 and 15 mM for *β*-CD and CM-*β*-CD, respectively, the solubility of cinnamaldehyde began to decrease, as all the inclusion complexes were formed.

### 3.2. Characterization of Modified-β-CD-ICs

Table 2 showed the dehydration yield (DY, %), encapsulation efficiency (EE, %), and CEO load (DL, mg/g) of the solid complexes of CEO with *β*-CD, Mal-, CM-, HP-, and DM-*β*-CD dehydrated by freeze-drying.

The DY of the samples increased in the following order: DM-*β*-CD < HP-*β*-CD < CM-*β*-CD < *β*-CD < Mal-*β*-CD. This could be attributed to the solubility of the different inclusion complexes according to the phase solubility results. The solid inclusion complexes of *β*-CD, Mal-*β*-CD, and CM-*β*-CD were visible before freezing, whereas those of HP-*β*-CD and DM-*β*-CD were not. DM-*β*-CD exhibited the highest CEO load value. From a toxicological and economic point of view, increasing the CEO load can be advantageous for the bulk formulation and bioavailability of bioactive compounds [27]. Additionally, the EE could be regarded as an indication of whether significant compound losses occurred during the dehydration process. Regarding the effect of the type of *β*-CDs on EE, significant differences (*p* > 0.05) were observed among the dehydrated inclusion complexes. The EE of the samples increased in the order: HP-*β*-CD < DM-*β*-CD < *β*-CD < Mal-*β*-CD < CM-*β*-CD. The lower EE for HP-ICs and DM-ICs probably resulted from easy dissolution, implying that cinnamaldehyde molecules and *β*-CD molecules are constantly in inclusion and dissociation. The CEO may be lost during the long-term preparation process. Furthermore, the different substituent groups of *β*-CDs may cause various steric hindrance, which hinders the insertion of CEO into the cavities of *β*-CDs.

### 3.3. Particles Morphology

**Figure 3:**
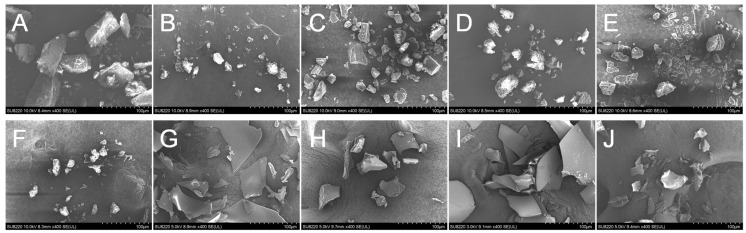
Scanning Electron Microscopy (SEM) images of *β*-CD (**A**), *β*-CD-ICs (**B**), Mal-*β*-CD (**C**), Mal-ICs (**D**), CM-*β*-CD (**E**), CM-ICs (**F**), HP-*β*-CD (**G**), HP-ICs (**H**), DM-*β*-CD (**I**), and DM-ICs (**J**) at 500× magnification.

The morphological features of Mal-*β*-CD, CM-*β*-CD, HP-*β*-CD, DM-*β*-CD, *β*-CD, and their inclusion complexes were observed via SEM (Figure 3).

Most *β*-CD-ICs, CM-*β*-CD-ICs, and Mal-*β*-CD-ICs presented as the small aggregates with many particles in a loose state (Figure 3B,D,F), whereas the corresponding *β*-CD appeared in a compact solid state with various sizes as irregularly shaped blocky particles (Figure 3A,C,E). Some small particles were still stuck to the surfaces of the large blocky solids. It can be seen that the *β*-CD-ICs, CM-ICs, and Mal-ICs showed irregular particles wherein the unique morphology of host molecules disappeared and loose aggregates were observed. The results are consistent with those of previous studies [28,29] and could result from the interference of guest molecules during the formation of hydrogen bonds.

In contrast, HP- and DM-*β*-CD presented as large and plate-like solids (Figure 3G,I), whereas their corresponding inclusion complexes were compact and small aggregates. The morphological features of the modified-*β*-CD-ICs were entirely dissimilar from those of the modified *β*-CD molecules, thus confirming the successful preparation of *β*-CD-, Mal-, CM-, HP-, and DM-ICs.

### 3.4. Molecular Docking Studies

**Figure 4:**
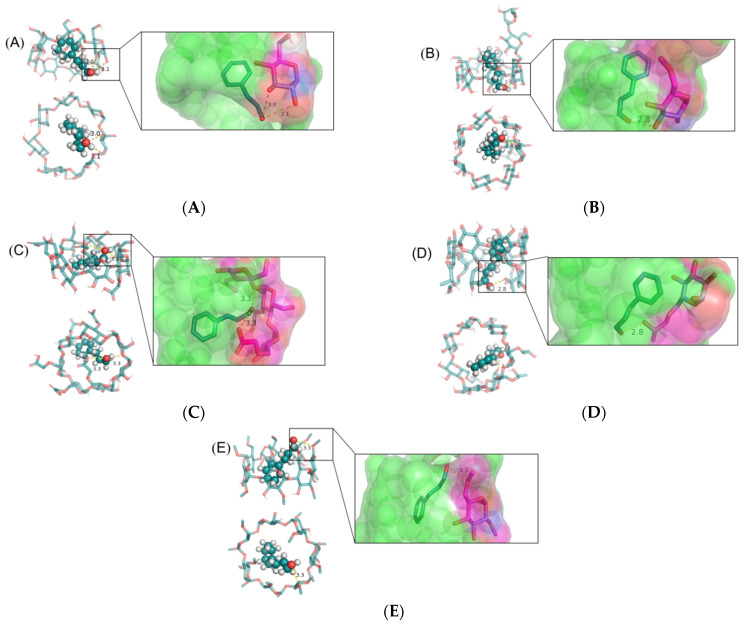
Docked images of cinnamaldehyde over *β*-CD (**A**), Mal-*β*-CD (**B**), CM-*β*-CD (**C**), HP-*β*-CD (**D**), and DM-*β*-CD (**E**) obtained from molecular docking study.

The cavities of *β*-CD and various modified *β*-CDs form cylindrical structures that entrap or load guest molecules. The optimal binding mode between cinnamaldehyde and various modified *β*-CDs was calculated via molecular modeling of the host-guest inclusion complexes (Figure 4). *β*-CD and its modified compounds encapsulated the acrolein unit of cinnamaldehyde and the aromatic ring stacked on the edge of the cavity, facilitating the formation of hydrogen bonds (yellow dashed line) with the hydroxyl group [30]. The inter-atomic distances between the interaction groups were 3.0 and 3.1 Å for *β*-CD-ICs, 2.8 Å for Mal-ICs, 3.3 Å and 3.3 Å for CM-ICs, 2.8 Å for HP-ICs, and 3.3 Å for DM-ICs, indicating possible H-bonds between cinnamaldehyde and various *β*-CDs. These interactions included H-bonding and hydrophobic interactions between the host (modified *β*-CD) and guest (cinnamaldehyde) molecules, which played a key role in the stability of *β*-CD, Mal-, CM-, HP-, and DM-ICs. The binding energies of *β*-CD, Mal-, CM-, HP-, and DM-*β*-CD conformations were −3.92, −4.42, −4.11, −4.72, and −3.7 kcal/mol, respectively. The above results confirmed the formation of thermodynamically stable inclusion complexes between *β*-CDs and cinnamaldehyde, as a higher negative docking binding energy indicates a more favorable complexation process [31].

**Figure 5:**
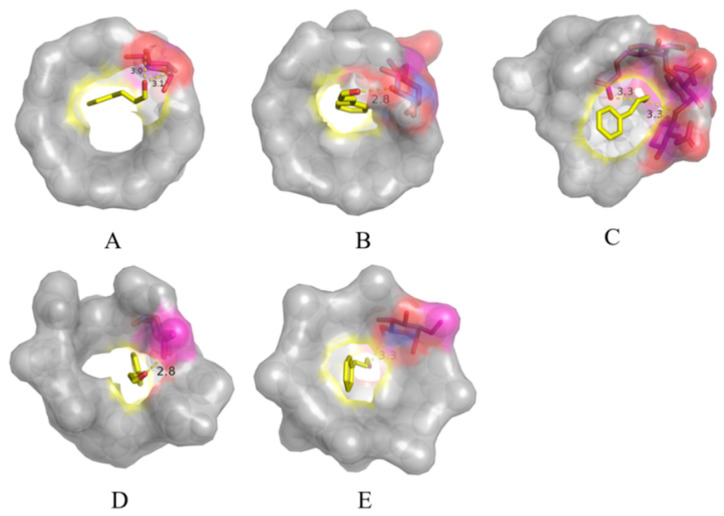
Vertical view images of cinnamaldehyde over *β*-CD (**A**), Mal-*β*-CD (**B**), CM-*β*-CD (**C**), HP-*β*-CD (**D**), and DM-*β*-CD (**E**) obtained from molecular docking study.

Figure 4 and Figure 5 illustrate the best binding conformations for cinnamaldehyde and various *β*-CDs, showing that the cinnamaldehyde molecule was completely encapsulated in the cavity of cyclodextrin. Zhang et al. [32] reported that two types (type A and type B) of inclusion complexes can be formed for *β*-CD or its modified compounds based on the orientation of the guest molecule; type A is more stable and has a lower binding energy than type B. The inclusion of *β*-CD, Mal-, and HP-*β*-CD with cinnamaldehyde belongs to type A, in which the benzene ring is close to the primary rim (small opening end). These three *β*-CDs had regular structures with little difference in the cavity opening area and it was shown that the benzene ring was located at the primary rim, whereas the acrolein group was located at the secondary rim (the large opening end). As shown in Figure 4B,D, the molecular geometric center of cinnamaldehyde was very close to the center of the Mal- and HP-*β*-CD cavities. For Mal-*β*-CD, the maltosyl group formed a prominent part with a large polarity; however, the protruding part extended slightly to the outside without blocking the entrance of the cyclodextrin cavity owing to the steric effects. Therefore, linear cinnamaldehyde molecules can be easily inserted into the cavity to form inclusion complexes. Additionally, for HP-*β*-CD, the benzene ring of the cinnamaldehyde molecule was located at the center of the internal cavity of cyclodextrin and the acrolein unit extended along the direction of 2-hydroxypropyl. The substitution of 2-hydroxypropyl increased the internal cavity volume of cyclodextrin and the possible formation of two hydrogen bonds causes the conformation to be more stable and result in a smaller binding energy.

In contrast, the inclusion of CM- and DM-*β*-CDs belongs to type B, which is characterized by the benzene ring located at the secondary rim. The substitution of carboxymethyl groups in CM-*β*-CD induced an irregular surface shape in the primary and secondary rims. It can be noticed that the substituent hindered the entrance of cinnamaldehyde molecule into the cavity of CM-*β*-CD owing to the steric hindrance and thus the benzene ring was located at the opposite rim (secondary rim). The binding energy of CM-*β*-CD (−4.11 kcal/mol) was lower than that of DM-*β*-CD (−3.7 kcal/mol). This could be attributed to the formation of two hydrogen bonds, which stabilized the structure and reduced the binding energy. Additionally, cinnamaldehyde was inclined in the cavity of cyclodextrin and the benzene ring and acrolein group units shrank in the hydrophobic cavity, leading to a more compact conformation, which may contribute to the lower binding energy of CM-*β*-CD compared to *β*-CD. For DM-*β*-CD, the substitution of a methyl group at the primary rim destroyed the stable secondary structure formed by the hydroxyl group, resulting in a reduced area of the primary rim, and the benzene ring was forced to the secondary rim owing to steric hindrance. Although the secondary rim of DM-*β*-CD was also substituted by methyl, it only replaced the hydrogen on one of the hydroxyl groups, whereas the other hydroxyl group was not affected and still maintained the opening state [33].

### 3.5. Volatile Chemical Composition of Modified-β-CD-ICs

The volatile constituents of CEO and its inclusion complexes were determined via GC-MS. Each component was determined with reference to a standard spectrum and the related literature [34]. The relative content of each component was calculated using peak area normalization. Table 3 presents the changes in CEO constituents before and after encapsulation.

A total of 39 compounds were identified in CEO via GC-MS (Table 3). (E)-Cinnamaldehyde (82.31%) and 2-methoxycinnamaldehyde (9.20%) were the two main components of CEO. The other components included (2-nitroprop-1-en-1-yl)-benzene (0.97%), benzaldehyde (0.86%), coumarin (0.85%), and α-pinene (0.77%). 

After encapsulation, eight compounds found in CEO were identified in *β*-CD-ICs whereas 15, 6, 9, and 26 compounds were identified in Mal-Ics, CM-Ics, HP-Ics, and DM-Ics, respectively. The main volatile components in all the inclusions were €-cinnamaldehyde and 2-methoxycinnamaldehyde. Particularly, myristic acid (9.28%), pentadecanoic acid (5.88%), and palmitic acid (21.02%) were identified as the main volatile components for *β*-Cd-ICs; palmitic acid (2.19%), and cinnamyl acetate (1.41%) for Mal-ICs; pentadecanoic acid (3.39%), and palmitic acid (9.77%) for CM-ICs; and benzenepropanal (1.29%) for HP-ICs, and cinnamyl acetate (1.47%) for DM-ICs. A few alcohols, terpenes, and alkanes compounds in CEOs disappeared after encapsulation. The relative content of CEO after encapsulation was lower than that found before encapsulation, for example, the total terpene content in *β*-CD-ICs, CM-ICs, and HP-ICs reduced from 3.23% to 0% after encapsulation. Furthermore, the total terpene content of Mal-ICs and DM-ICs reduced from 3.23% to 0.40% and 0.90%, respectively. The relative alcohol content of all the modified-*β*-CD-ICs was also reduced. However, the contents of several components increased; the benzenepropanal content of HP-ICs and DM-ICs increased from 0.64% to 1.29% and 0.83%, respectively, and the 2-methoxycinnamaldehyde content increased from 9.20% to 13.63% for Mal-ICs. This result is consistent with previous reports on encapsulation of *β*-CDs [35] and could be attributed to the selective encapsulation of different CEO components in cyclodextrin, in which the contents of some components decreased or increased during the embedding process. It can be noticed that DM-ICs had the most diverse chemical composition, implying that DM-*β*-CD is more suitable for the encapsulation of CEO. It can encapsulate the compounds of CEO as much as possible when they are lost during the preparation process. This result is consistent with the CEO load (Table 2). Moreover, of all the components, HP-*β*-CD was preferred to encapsulate cinnamaldehyde. The HP-ICs had the highest relative content of cinnamaldehyde for all the tested cyclodextrin, which means it has the greatest adaptability of HP-*β*-CD to cinnamaldehydes. This is consistent with the highest negative docking binding energy, which indicated that the combination of HP-*β*-CD and cinnamaldehyde had the greatest affinity and the most stable conformation. These differences in modified cyclodextrin encapsulation may be used as criteria for preparing ideal inclusion complexes.

The biological activity of the EOs is primarily attributed to the presence of volatile compounds such as terpenes, alcohols, acids, esters, and aldehydes [36,37]. Therefore, changes in the volatile component content affect their antibacterial capacity. Terpenes and terpenoids are the main antibacterial compounds in EOs, followed by polyphenols [38]. They exert antibacterial activity through synergistic and additive effects with other compounds [39]. Specifically, cinnamaldehyde disturbs the interaction with bacterial cell membranes via interference in the glycerophospholipid biosynthesis pathway of food-borne pathogens [40,41] and is regarded as the main component with potent antibacterial activity. Generally, the loss of some terpene and terpenoid compounds and the reduction in the content of other compounds after encapsulation may cause a decrease in the biological performance of CEO.

### 3.6. Antioxidant Activities of Modified-β-CD-ICs

**Figure 6:**
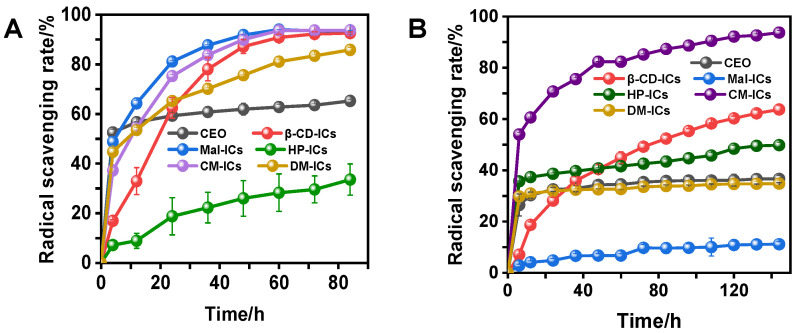
Radical scavenging of CEO and various CD-ICs with (**A**) 1,1-diphenyl-2-picrylhydrazyl (DPPH) method and (**B**) phenyl-4,4,5,5-tetramethylimidazoline-1-oxyl-3-oxide (PTIO) method.

The results of different assays (FRAP, DPPH and PTIO) to determine the antioxidant activities of modified-*β*-CD-ICs and CEO are presented in Table 4 and Figure 6A,B. As shown in Table 4, CEO exhibited the highest reducing power (FRAP) of 1.075 ± 0.024 μmol Trolox/mg and the reducing capacity of CD-ICs decreased in the following order: Mal-ICs > CM-ICs > DM-ICs > *β*-CD-ICs > HP-ICs. To exclude the interference of cyclodextrin on antioxidant activity, free *β*-CDs were also subjected to the same measurement. The results (Table 5) showed that the reducing power of free *β*-CDs was weak and negligible (DPPH and PTIO).

At the beginning of the reaction (0–4 h), the DPPH scavenging ability of all the samples decreased in the following order: CEO > Mal-ICs > DM-ICs > CM-ICs > *β*-CD-ICs > HP-ICs. Similarly, the PTIO scavenging ability of all the samples decreased in the following order: CM-ICs > CEO > HP-ICs > DM-ICs > *β*-CD-ICs > Mal-ICs. These results are consistent with the antioxidant activity of HP-*β*-CD inclusion complexes reported by Kamimura et al. [42] and *β*-CD inclusion complexes reported by Santos et al. [43]. The encapsulation of EOs have weakened antioxidant activity. When encapsulated in Mal-, DM-, and CM-*β*-CDs, CEOs maintained similar reducing power and antioxidant activity of scavenging DPPH as free CEOs, whereas HP- and DM-*β*-CD maintained the antioxidant activity of scavenging PTIO. It can be noticed that CM-ICs had stronger DPPH and PTIO scavenging activities (Figure 6A,B) than CEO (after 24 h). This was probably because scavenging PTIO is a long process, which means PTIO radicals cannot be completely scavenged in a short time. CEO may decrease during the scavenging process due to its chemical instability, which means the better the protection for CEOs, the stronger scavenging effects. CM-*β*-CD is the best shell material to protect CEO in an aqueous solution. However, when CEO was encapsulated in HP-*β*-CD and *β*-CD, the reducing power and antioxidant activity of the DPPH scavenging were significantly reduced. The ability to scavenge PTIO was also clearly reduced when CEO was encapsulated in Mal-*β*-CD and *β*-CD. We speculate that the partial disappearance of volatile compounds and the reduction in the relative content of some CEO components after encapsulation decreased the antioxidant performance of CEO, thereby decreasing the antioxidant ability of most inclusion complexes. The CM-ICs presented an increase in reducing power and maintained the scavenging ability of PTIO, which could be attributed to the fact that the PTIO and FRAP assays reacted in the water-soluble system. The poor water solubility of EOs hinders their antioxidant ability. It should be pointed out that DM- and HP-ICs can dissolve in water completely, which is similar to CEO, whereas Mal-, CM-, and *β*-CD-ICs were partially or hardly dissolved. Therefore, the low solubility inclusion complexes presented a higher scavenging ability owing to the protection of CEO through dissolution equilibrium.

With increasing reaction time, the DPPH scavenging rate of CEO saturated within 4 h. However, the DPPH scavenging rate of the modified-*β*-CD-ICs increased rapidly and then slowly. This suggests that the encapsulation of CEOs in CDs could scavenge DPPH in a slow and gradual process, as the liquid core materials can be constantly released into the external environment through dissolution and diffusion, which is consistent with a previous report [29]. Similarly, the PTIO scavenging rates of CEO, HP-ICs, DM-ICs, and Mal-ICs were nearly saturated within 6 h, whereas those of CM-ICs and *β*-CD-ICs exhibited a slowly increasing trend, indicating that the stability of the guest molecule improved during the complexing process such that they could react with PTIO completely. Notably, *β*-CD-ICs presented the best stable slow-release characteristics for each type of radical. These results could be attributed to the same inclusion mode (Type B) for CM-ICs and *β*-CD-ICs, which were in an insoluble state in the aqueous solution. Particularly, *β*-CD has a rather rigid structure among all the modified *β*-CDs [6]. The difference in the slow-release effect between DPPH and PTIO scavenging for various ICs could be attributed to the solubility of the CDs. The complete dissolution of DM- and HP-ICs caused a decrease in the slow release. Moreover, the radical scavenging of most ICs clearly increased at the end of the reaction, which was higher than that of CEO. This phenomenon could be attributed to the encapsulation effect of CDs on free radicals in the solution after a long reaction time. A study [44] has shown that *β*-CD can selectively bind aromatic compounds with suitable shapes and sizes to form a supramolecular system. DPPH and PTIO, which are aromatic derivative compounds, were probably partially complexed in various *β*-CDs during a long-time scavenging process, resulting in an increase in the scavenging rate; thus, CDs had radical scavenging capacity in appearance but no actual antioxidant effect. It can be concluded that CM-*β*-CD and Mal-*β*-CD were the most suitable shell materials in water-soluble and alcohol-soluble systems, respectively, among all the tested cyclodextrins owing to their excellent antioxidant properties and persistence in the reaction system.

### 3.7. MIC of CEO and Modified-β-CD-ICs

**Figure 7:**
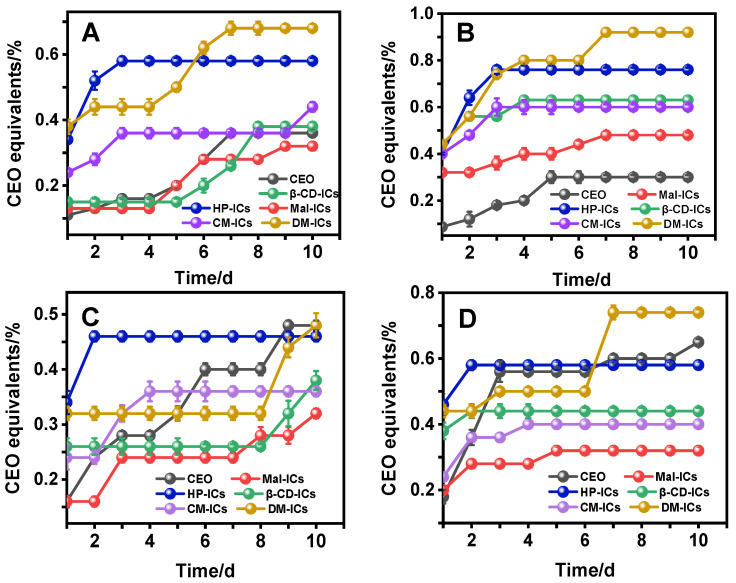
MIC values of CEO and modified-*β*-CD-ICs against (**A**) E. coli, (**B**) S. aureus, (**C**) Salmonella, (**D**) P. aeruginosa (%, *w*/*w*).

MIC indicates the antibacterial capacity of the test samples. A smaller value represents stronger antibacterial ability [1]. Figure 7 presents the change in MIC values of CEO and CEO-modified-*β*-CD-ICs during the 10-day period.

CEOs displayed antibacterial activity against all the tested bacteria, including Gram-positive (Figure 7B) and Gram-negative (Figure 7A,C,D) bacteria in a 10-day storage period, and CEO exhibited the strongest antibacterial ability on the first day (*p* ≤ 0.05). The minimum MIC values of CEO were observed against *S. aureus* (0.087 ± 0.018%, *w*/*w*), *E. coli* (0.11 ± 0.003%), *Salmonella* (0.16 ± 0.011%), and *P. aeruginosa* (0.18 ± 0.021%). This result is consistent with a previous report [45] that CEO is highly effective against *S. aureus*. The antibacterial effect of the samples at the beginning of the period (the first day) decreased in the following order: CEO > Mal-ICs > *β*-CD-ICs > CM-ICs > HP-ICs > DM-ICs for *E. coli*, CEO > Mal-ICs > HP-ICs > CM-ICs > DM-ICs > *β*-CD-ICs for *S. aureus*, and CEO > Mal-ICs > CM-ICs > *β*-CD-ICs > DM-ICs > HP-ICs for *Salmonella* and *P. aeruginosa*. Cristian [46] reported a decrease in the antibacterial capacity of coriander EO encapsulated in *β*-CD compared to free EO and Kfoury [47] determined that the antifungal activity of phenylpropanoids decreased after encapsulation in HP-*β*-CDs. However, for Mal-*β*-CD, CEO encapsulation maintained antimicrobial activity against *E. coli*, *Salmonella*, and *P. aeruginosa*, which was close to that of the free CEO. The strong antimicrobial activity of Mal-ICs could be explained as follows: (1) as shown in Figure 2, Mal-ICs were low-solubility inclusion complexes, which were partially dissolved in nutrient broth initially for directly inhibiting bacteria and the undissolved part of Mal-ICs was fixed in agar for continued antibacterial activity; (2) as revealed by GC-MS (Table 3), the Mal-ICs contained more aldehydes, terpenes, and alcohols than the other tested cyclodextrins, which maintain the antibacterial properties of CEO as much as possible after encapsulation.

With increased storage time, the antibacterial effect of CEO and modified-*β*-CD-ICs were significantly weakened. For *E. coli,* Mal-ICs demonstrated stronger antibacterial properties than CEO. For *Salmonella* and *P. aeruginosa*, Mal-ICs, CM-ICs, *β*-CD-ICs, and HP-ICs demonstrated stronger antibacterial properties than did CEO. Thus, the hierarchy in antibacterial capacity of modified-*β*-CD-ICs against *E. coli*, *Salmonella*, and *P. aeruginosa* (after the seventh day) was as follows: Mal-ICs > CM-ICs > *β*-CD-ICs > HP-ICs > DM-ICs. These results could be ascribed to the loss of volatile components of the CEO. Yang et al. [1] and Angane et al. [38] proposed that the antibacterial ability of EOs can be attributed to terpenes, alcohols, and aldehydes and that the changes in the volatile component content would affect the antibacterial properties. Additionally, the retention of volatile components was positively correlated with the antibacterial ability of EO-nanoemulsions during a 56-day storage period [48]. In this study, modified *β*-CD was utilized to protect the volatile constituents of CEO, consequently the strong antibacterial properties of CEO retained for a long time. 

The antibacterial effect of Mal-, CM-, and *β*-CD-ICs was much greater than that of DM- and HP-ICs. This could be attributed to the different inclusion modes in which DM- and HP-ICs were completely dissolved when dispersed in a liquid medium, whereas Mal-, CM-, and *β*-CD-ICs were dissolved in a small amount or were even insoluble in nutrient broth. Therefore, low-solubility inclusion complexes can be uniformly dispersed in a solid medium to provide long-term and sustained antibacterial effects owing to their better slow-release properties. Moreover, for B_s_ and B_I_ low-solubility inclusion complexes, the long-term antibacterial effect was positively correlated with aldehyde content as shown by GC-MS (Table 3), which played a major role in the antibacterial ability of inclusion complexes. In addition, *β*-CD, HP-*β*-CD, and DM-*β*-CD had symmetric structures and an even distribution of hydroxyl groups, leading to the formation of hydroxyl groups in the cavity to form a secondary structure and preventing reaction with the external environment. It has been reported that hydroxyl has an inevitably close relationship with antibacterial activities, which decrease or disappear after the hydroxyl group is removed [49]. For Mal-*β*-CD, except for the hydroxyl group in the cavity, the maltosyl branched chain outside the cavity also had hydroxyl groups that did not form intermolecular hydrogen bonds with the hydroxyl groups in the cavity, thus it was free to react. For CM-*β*-CD, the substitution of carboxymethyl enhanced its ability to dissociate protons, which reduced the pH in cells, inhibiting the basic metabolic reactions and achieve antibacterial effects [50].

However, CEO exhibited the strongest antibacterial activity against *S. aureus* during the 10-day storage period. It could be noticed that *S. aureus* is a Gram-positive bacteria, whereas *E. coli*, *Salmonella*, and *P. aeruginosa* are Gram-negative bacteria. Gram-positive bacterial are more susceptible to EOs than Gram-negative bacteria and this phenomenon could result from the direct interaction of the cell membrane with the hydrophobic components of EOs [51,52]. When Gram-positive bacteria are in direct contact with EOs, their membranes can combine with the EO, which causes a significant antibacterial effect. However, when EOs were encapsulated by *β*-CD, it was difficult for the membrane of *S. aureus* to directly contact the hydrophobic components of CEO, which means that it cannot show the antibacterial ability until the CEO is released slowly from the inclusion complexes. Therefore, CEO presented the strongest antibacterial effect against *S. aureus* during the 10-day storage period. In contrast, compared with the Gram-positive bacteria, Gram-negative bacteria possess a hydrophilic cell wall [53,54], and this feature is helpful for bacteria that have active contact with inclusion complexes. Therefore, Gram-negative bacteria were more easily influenced by CEO encapsulation in *β*-CDs and the antibacterial effect of partially modified-*β*-CD-ICs was stronger than that of CEO during long-term storage. It can be seen that pure CEO is more suitable to inhibit Gram-positive bacteria and Mal-*β*-CD is the best shell material to improve the long-term antibacterial ability of CEO against Gram-negative bacteria.

## 4. Conclusions

In conclusion, this study investigated the antioxidant and antibacterial properties of CD-CEO complexes. Depending on the different solubilities of the inclusion complexes, DM-*β*-CD and HP-*β*-CD were considered A-type whereas CM-*β*-CD, Mal-*β*-CD, and *β*-CD were B-type. The insertion of cinnamaldehyde into all the tested *β*-CDs cavities possibly occurred via hydrogen binding. It was observed that Mal-*β*-CD, CM-*β*-CD, and *β*-CD encapsulation of CEO significantly increased their antioxidant and antibacterial activities during long-term storage. In this regard, CM-ICs steadily presented higher antioxidant activity than free CEO for long-time use and Mal-ICs showed the best antibacterial ability for long-time storage, indicating that low-solubility inclusion complexes enhanced the continued antioxidant and antibacterial capacity.

## Figures and Tables

**Table 1:** Reaction parameter solid complexes by freeze-drying for CEO encapsulated with *β*-CD, Mal-, CM-, HP-, and DM-*β*-CD.

CD	Reaction Parameter		
	Ratio of Oil to Shell Materials, (*w*/*w*)	Time (h)	Temperature (°C)
*β*-CD	1:10	3	40
Mal-*β*-CD	1:10	1.5	15
CM-*β*-CD	1:8	2	30
HP-*β*-CD	1:10	1.5	30
DM-*β*-CD	1:12	1	50

**Table 2:** Reaction parameter and result of solid complexes by freeze-drying for CEO encapsulated with *β*-CD, Mal-, CM-, HP-, and DM-*β*-CD *^a^*.

CD	DY (%)	EE (%)	CEO Load (mg/g)
*β*-CD	87.33	57.37 ±1.23 ^c^	87.58 ±0.72 ^a^
Mal-*β*-CD	91.25	68.90 ±2.31 ^b^	75.86 ±1.63 ^c^
CM-*β*-CD	86.67	72.73 ±1.69 ^a^	84.57 ±0.19 ^b^
HP-*β*-CD	86.05	43.88 ±1.49 ^e^	34.82 ±0.82 ^d^
DM-*β*-CD	78.18	49.90 ±0.85 ^d^	88.35 ±2.05 ^a^

*^a^* Values are presented as the mean ± standard deviation. Means in the same column with different superscripts are significantly different (*p* ≤ 0.05).

**Table 3:** GC-MS analysis of five modified *β*-cyclodextrin *^a^*.

Serial Number	Compound Name -Molecular Formula	Relative Content (%)					
		CEO	CEO- *β*-CD	CEO-Mal-*β*-CD	CEO- CM-*β*-CD	CEO-HP-*β*-CD	CEO- DM-*β*-CD
1	Styrene-C_8_H_8_	0.146	-	-	-	-	-
2	*α*-Pinene-C_10_H_16_	0.769	-	-	-	-	0.024
3	Camphene-C_10_H_16_	0.060	-	-	-	-	0.033
4	Benzaldehyde-C_7_H_6_O	0.864	0.901	0.526	1.391	0.367	0.470
5	*β*-pinene-C_10_H_16_	0.033	-	-	-	-	0.020
6	p-Cymene-C_10_H_14_	0.042	-	-	-	-	0.027
7	Limonene-C_10_H_16_	0.036	-	-	-	-	0.017
8	Salicylal-C_7_H_6_O_2_	0.221	-	-	-	0.160	0.145
9	Acetophenone-C_8_H_8_O	0.039	1.080	-	-	-	-
10	Phenylethyl alcohol-C_8_H_10_O	0.471	-	-	-	-	0.037
11	Benzenepropanal-C_9_H_10_O	0.640	-	0.364	-	1.293	0.825
12	((1S)-endo)-(-)-borneol-C_10_H_16_O	0.133	-	0.416	-	-	-
13	*α*-Terpineol-C_10_H_18_O	0.031	-	-	-	-	0.028
14	2-Methoxybenzaldehyde-C_8_H_8_O_2_	0.667	1.032	0.646	-	0.754	0.689
15	Acetic acid 2-phenylethyl ester-C_10_H_12_O_2_	0.098	-	0.321	-	-	0.178
16	(E)-Cinnamaldehyde-C_9_H_8_O	82.306	51.005	77.241	76.712	91.941	85.026
17	3-Phenylprop-2-en-1-ol-C_9_H_10_O	0.112	-	-	-	-	-
18	Caryophyllene-C_15_H_24_	0.142	-	-	-	-	0.122
19	(E)-alpha-bergamotene-C_15_H_24_	0.074	-	-	-	-	0.094
20	Coumarin-C_9_H_6_O_2_	0.845	-	0.296	-	0.330	0.122
21	(2-Nitroprop-1-en-1-yl)benzene-C_9_H_9_NO_2_	0.973	-	-	-	-	-
22	2-Methoxycinnamaldehyde-C_10_H_10_O_2_	9.199	8.144	13.626	6.200	3.414	8.788
23	Gamma-muurolene-C_15_H_24_	0.189	-	-	-	-	-
24	*α*-curcumene-C_15_H_22_	0.158	-	-	-	-	0.229
25	*α*-muurolene-C_15_H_24_	0.146	-	-	-	-	-
26	*β*-bisabolene-C_15_H_24_	0.159	-	-	-	-	0.153
27	(+)-δ-cadinene-C_15_H_24_	0.320	-	-	-	-	0.211
28	Nerolidol-C_15_H_26_O	0.189	-	-	-	-	-
29	Spathulenol-C_15_H_24_O	0.163	-	-	-	-	-
30	Caryophyllene oxide-C_15_H_24_O	0.149	-	-	-	-	-
31	alpha-Bisabolol-C_15_H_26_O	0.062	-	-	-	-	0.177
32	Myristic acid-C_14_H_28_O_2_	0.095	9.277	1.175	2.540	-	0.304
33	Benzyl benzoate-C_14_H_12_O_2_	0.081	-	0.232	-	-	0.194
34	Pentadecanal-C_15_H_30_O	0.020	-	-	-	-	0.017
35	Pentadecanoic acid- C_15_H_30_O_2_	0.042	5.882	0.736	3.385	0.168	0.142
36	Phenethyl benzoate-C_15_H_14_O_2_	0.055	-	0.322	-	-	-
37	Palmitic acid-C_16_H_32_O_2_	0.241	21.024	2.191	9.772	1.064	0.458
38	Phytol-C_20_H_40_O	0.017	-	0.109	-	-	-
39	Anethole-C_10_H_12_O	0.015	-	0.397	-	-	-
40	Cinnamyl acetate-C_11_H_12_O_2_	-	1.655	1.408	-	0.509	1.469
Terpenes type		14 (3.23%)	0	1 (0.40%)	0	0	9 (0.90%)
Alcohols type		8 (1.18%)	0	2 (0.52%)	0	0	3 (0.25%)
Aldehydes type		7 (93.92%)	4 (61.09%)	5 (92.40%)	3 (84.30%)	6 (97.93%)	7 (95.96%)
Alkanes type		1 (0.04%)	0	0	0	0	1 (0.02%)
Fatty acids type		3 (0.38%)	3 (36.19%)	3 (4.10%)	3 (15.70%)	2 (1.23%)	3 (0.90%)
Esters type		4 (1.08%)	1 (1.655%)	5 (2.58%)	0	2 (0.84%)	4 (1.97%)

*^a^* Note: “-”(<0.01%).

**Table 4:** The reducing power of the CEO, *β*-CD-ICs, Mal-ICs, HP-ICs, CM-ICs, and DM-ICs by using FRAP method *^a^*.

Sample	CEO	*β*-CD-ICs	Mal-ICs	HP-ICs	CM-ICs	DM-ICs
Trolox equivalents (μmol Trolox /mg)	1.075 ± 0.024 ^ab^	0.752 ± 0.079 ^c^	1.092 ± 0.016 ^a^	0.532 ± 0.033 ^d^	1.065 ± 0.054 ^ab^	0.979 ± 0.021 ^b^

*^a^* Values are presented as the mean ± standard deviation. Mean values in the same row followed by different letters (a-d) indicate statistically significant (*p* < 0.05) differences among samples.

**Table 5:** The reducing power of *β*-CD, Mal-*β*-CD, HP-*β*-CD, CM-*β*-CD, and DM-*β*-CD by using FRAP method *^a^*.

Sample	*β*-CD	Mal-*β*-CD	HP-*β*-CD	CM-*β*-CD	DM-*β*-CD
Trolox equivalents (μmol Trolox /mg)	0.008 ±0.001 ^bc^	0.018 ±0.001 ^b^	0.119 ±0.009 ^a^	0.003 ±0.001 ^c^	0.018 ±0.003 ^b^

*^a^* Values are presented as the mean ± standard deviation. Mean values in the same row followed by different letters (a–d) indicate statistically significant (*p* < 0.05) differences among samples.

## Data Availability

Data is contained within the article.

## Supplementary Materials

The following supporting information can be downloaded at: https://www.mdpi.com/article/10.3390/foods12010045/s1, Figure S1: Effect of the (A) ratio of oil to wall materials, (B) time, (C) temperature on the EE of *β*-CD-ICs.; Figure S2. Effect of the (A) ratio of oil to wall materials, (B) time, (C) temperature on the EE of Mal-ICs.; Figure S3. Effect of the (A) ratio of oil to wall materials, (B) time, (C) temperature on the EE of CM-ICs.; Table S1: The design and results of the orthogonal formulation of *β*-CD-ICs *^a^*; Table S2. The design and results of the orthogonal formulation of Mal-ICs ^a^; Table S3. The design and results of the orthogonal formulation of CM-ICs ^a^; Figure S4. Effect of the (A) ratio of oil to wall materials, (B) time, (C) temperature on the EE of HP-ICs; Table S4. The design and results of the orthogonal formulation of HP-ICs ^a^; Figure S5. Effect of the (A) ratio of oil to wall materials, (B) time, (C) temperature on the EE of DM-ICs; Table S5. The design and results of the orthogonal formulation of DM-ICs ^a^.

## Abbreviations

*β*-CD-ICs: *β*-cyclodextrin cinnamon essential oil inclusion complexes, Mal-ICs: maltosyl-*β*-cyclodextrin cinnamon essential oil inclusion complexes, CM-ICs: O-carboxymethyl-*β*-cyclodextrin cinnamon essential oil inclusion complexes, HP-ICs: hydroxypropyl-*β*-cyclodextrin cinnamon essential oil inclusion complexes, DM-ICs: (2,6-di-o-methyl)-*β*-cyclodextrin cinnamon essential oil inclusion complexes, GC-MS: Gas chromatography-mass spectrometer.
