# Supplementary material for: Encapsulation Efficiency and Functional Stability of Cinnamon Essential Oil in Modified β-cyclodextrins: In Vitro and In Silico Evidence
Source: Foods. 2022 Dec 22;12(1):45. doi: 10.3390/foods12010045 (PMC9818807; doi:10.3390/foods12010045)
Supplement: Supplementary file 1 [file foods-12-00045-s001.zip › foods-2030792-supplementary.pdf]

Supplementary Information

Encapsulation Efficiency and Functional Stability of Cinnamon Essential Oil in Modified β-Cyclodextrins: In Vitro and In Silico Evidence

Kegang Wu, Tong Zhang *, Xianghua Chai, Xuejuan Duan, Dong He, Hongpeng Yu, Xiaoli Liu and Zhihua Tao

School of Chemical Engineering and Light Industry, Guangdong University of Technology, Guangzhou, 511443, China
* Correspondence: fsezzz308364445@163.com

S1. Experimental methods

S1.1. The effects of the preparation conditions on the EE of β-CD-ICs

The *β*-CDs solution was prepared using hot distilled water with water: *β*-CD ratio of 9:1. CEO solution was prepared using absolute alcohol. The CEO solution was injected into *β*-CDs solution.

The *β*-CD-ICs preparation conditions were controlled to evaluate the influences of the oil to wall ratio (1:4, 1:6, 1:8, 1:10, and 1:12) on the EE at 60 °C and 300 rpm for 2 h, the time (0.5, 1.0, 1.5, 2.0, 2.5, and 3.0 h) on the EE with the oil ratio of 1:10 at 60 °C and 300 rpm, and the temperature (30, 40, 50, 60, and 70 °C) on the EE with the oil ratio of 1:10 at 300 rpm for 2.5 h.

S1.2. The effects of the preparation conditions on the EE of Mal-ICs

The Mal-ICs preparation conditions were controlled to evaluate the influences of the oil to wall ratio (1:8, 1:10, 1:12, 1:14, and 1:16) on the EE at 30 °C and 300 rpm for 2 h, the time (0.5, 1.0, 1.5, 2.0, 2.5, and 3.0 h) on the EE with the oil/wall ratio of 1:12 at 30 °C and 300 rpm, and the temperature (15, 20, 25, 30, 35, and 40 °C) on the EE with the oil ratio of 1:12 at 300 rpm for 1.5 h.

S1.3. The effects of the preparation conditions on the EE of CM-ICs

The CM-ICs preparation conditions were controlled to evaluate the influences of the oil to wall ratio (1:6, 1:8, 1:10, 1:12, 1:14, and 1:16) on the EE at 30 °C and 300 rpm for 2 h, the time (0.5, 1.0, 1.5, 2.0, 2.5, and 3.0 h) on the EE with the oil/wall ratio of 1:16 at 30 °C and 300 rpm, and the temperature (15, 20, 25, 30, 35, and 40 °C) on the EE with the oil ratio of 1:16 at 300 rpm for 2 h.

S1.4. The effects of the preparation conditions on the EE of HP-ICs

The HP-ICs preparation conditions were controlled to evaluate the influences of the oil to wall ratio (1:4, 1:6, 1:8, 1:10, and 1:12) on the EE at 30 °C and 300 rpm for 2 h, the time (0.5, 1.0, 1.5, 2.0, and 2.5 h) on the EE with the oil/wall ratio of 1:8 at 30 °C and 300 rpm, and the temperature (20, 30, 40, 50, and 60 °C) on the EE with the oil ratio of 1:8 at 300 rpm for 1 h.

S1.5. The effects of the preparation conditions on the EE of DM-ICs

The DM-ICs preparation conditions were controlled to evaluate the influences of the oil to wall ratio (1:4, 1:6, 1:8, 1:10, 1:12, and 1:14) on the EE at 30 °C and 300 rpm for 2 h, the time (0.5, 1.0, 1.5, 2.0, 2.5, and 3.0 h) on the EE with the oil/wall ratio of 1:12 at 30 °C and 300 rpm, and the temperature (20, 30, 40, 50, and 60 °C) on the EE with the oil ratio of 1:12 at 300 rpm for 2 h.

S2. Effects of preparation conditions on the encapsulation efficiency (EE)

S2.1. β-CD-ICs

Figure S1 (A) showed that the EE of *β*-CD-ICs reached the highest yield at the oil/wall material mass ratio of 1:10 (w/w). **Figure S1** (B) showed that the EE of *β*-CD-ICs reached the highest yield at 2.5 h. **Figure S1** (C) showed that the EE of *β*-CD-ICs reached the highest yield at 50 °C.

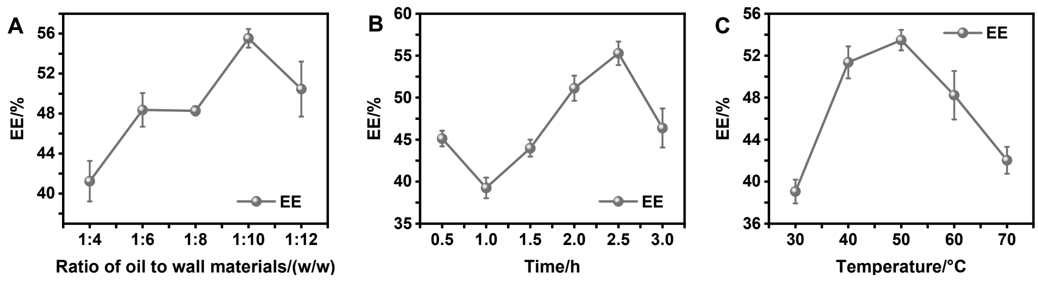

Figure S1. Effect of the (A) ratio of oil to wall materials, (B) time, (C) temperature on the EE of *β*-CD-ICs.

As shown in **Table S1**, when the mass ratio of oil to wall materials, time, and temperature conditions were 1:10 (w/w), 3 h, and 40 °C, respectively, the EE reached the highest value.

Table S1. The design and results of the orthogonal formulation of *β*-CD-ICs [a]

Numbers	Factors				EE%
	A	B	C	D	
1	1（1:8）	1（2h）	1（40°C）	1	56.90 ± 2.31
2	1	2（2.5h）	2（50°C）	2	42.96 ± 1.23
3	1	3（3h）	3（60°C）	3	47.68 ± 4.20
4	2（1:10）	1	2	3	37.12 ± 1.32
5	2	2	3	1	38.52 ± 1.38
6	2	3	1	2	57.37 ± 1.23
7	3（1:12）	1	3	2	45.39 ± 1.76
8	3	2	1	3	55.68 ± 2.87
9	3	3	2	1	52.62 ± 1.90
k_1	49.18	46.47	56.65	49.35	
k_2	44.34	45.72	44.23	48.57	
k_3	51.23	55.56	43.86	46.83	
R	6.89	9.84	12.79	2.52	

[a] Note: A means the ratio of oil to wall materials, B means the time value, C means the temperature values, and D means blank column. k_1 is the mean values for level 1 of each factor; k_2 is the mean values for level 2 of each factor; k_3 is the mean values for level 3 of each factor. R is the difference between the maximum and minimum of the encapsulation efficiency (EE) for every level. R = max (k_i) − min (k_i).

S2.2. Mal-ICs

Figure S2 (A) showed that the EE of Mal-ICs reached the highest yield at the oil/wall material mass ratio of 1:12 (mol/mol). **Figure S2** (B) showed that the EE of Mal-ICs reached the highest yield at 1.5 h. **Figure S2** (C) showed that the EE of Mal-ICs reached the highest yield at 15 °C.

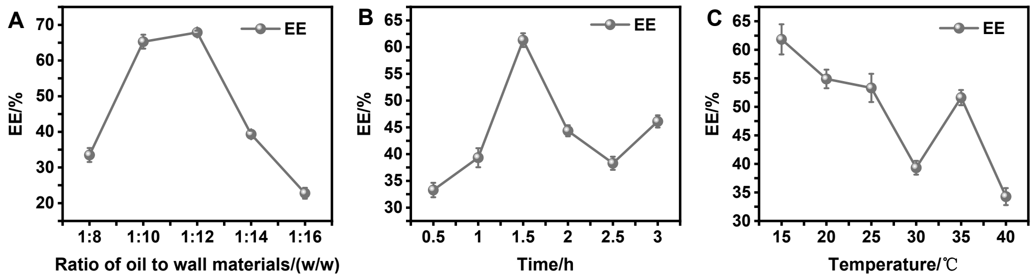

Figure S2. Effect of the (A) ratio of oil to wall materials, (B) time, (C) temperature on the EE of Mal-ICs.

As shown in **Table S2**, when the mass ratio of oil to wall materials, time, and temperature conditions were 1:10 (w/w), 1 h, and 80 °C, respectively, the EE reached the highest value.

Table S2. The design and results of the orthogonal formulation of Mal-ICs [a]

Numbers	Factors				EE%
	A	B	C	D	
1	1（1:10）	1（1.5h）	1（15°C）	1	68.90 ± 2.31
2	1	2（2h）	2（20°C）	2	42.96 ± 1.23
3	1	3（3h）	3（25°C）	3	47.68 ± 4.20
4	2（1:12）	1	2	3	37.12 ± 1.32
5	2	2	3	1	38.52 ± 1.38
6	2	3	1	2	35.56 ± 1.12
7	3（1:14）	1	3	2	45.39 ± 1.76
8	3	2	1	3	55.68 ± 2.87
9	3	3	2	1	52.62 ± 1.90
k_1	53.18	50.47	53.38	53.35	
k_2	37.06	45.72	44.23	41.30	
k_3	51.23	45.28	43.86	46.82	
R	14.16	5.19	9.52	12.04	

[a] Note: A means the ratio of oil to wall materials, B means the time value, C means the temperature values, and D means blank column. k_1 is the mean values for level 1 of each factor; k_2 is the mean values for level 2 of each factor; k_3 is the mean values for level 3 of each factor. R is the difference between the maximum and minimum of the encapsulation efficiency (EE) for every level. R = max (k_i) − min (k_i).

S2.3. CM-ICs

Figure S3 (A) showed that the EE of CM-ICs reached the highest yield at the oil/wall material mass ratio of 1:16 (mol/mol). **Figure S3** (B) showed that the EE of CM-ICs reached the highest yield at 2 h. **Figure S3** (C) showed that the EE of CM-ICs reached the highest yield at 30 °C.

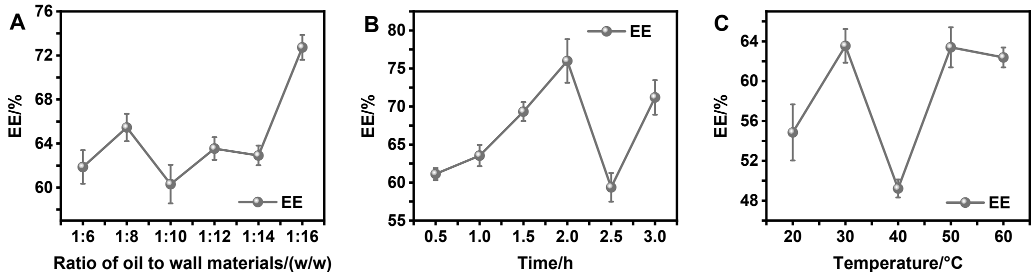

Figure S3. Effect of the (A) ratio of oil to wall materials, (B) time, (C) temperature on the EE of CM-ICs.

As shown in **Table S3**, when the mass ratio of oil to wall materials, time, and temperature conditions were 1:8 (w/w), 2 h, and 30 °C, respectively, the EE of CM-ICs reached the highest value.

Table S3. The design and results of the orthogonal formulation of CM-ICs [a]

Numbers	Factors				EE%
	A	B	C	D	
1	1（1:8）	1（1.5h）	1（20°C）	1	68.90 ± 2.31
2	1	2（2h）	2（30°C）	2	72.73 ± 1.69
3	1	3（3h）	3（50°C）	3	47.68 ± 4.20
4	2（1:12）	1	2	3	39.12 ± 1.32
5	2	2	3	1	71.52 ± 0.83
6	2	3	1	2	53.26 ± 1.17
7	3（1:16）	1	3	2	45.39 ± 1.76
8	3	2	1	3	55.68 ± 2.87
9	3	3	2	1	62.18 ± 1.97
k_1	63.10	51.14	59.28	67.53	
k_2	54.63	66.64	57.13	57.13	
k_3	54.42	54.37	54.86	47.49	
R	8.68	15.5	4.42	20.04	

[a] Note: A means the ratio of oil to wall materials, B means the time value, C means the temperature values, and D means blank column. k_1 is the mean values for level 1 of each factor; k_2 is the mean values for level 2 of each factor; k_3 is the mean values for level 3 of each factor. R is the difference between the maximum and minimum of the encapsulation efficiency (EE) for every level. R = max (k_i) − min (k_i).

S2.4. HP-ICs

Figure S4 (A) showed that the EE of HP-ICs reached the highest yield at the oil/wall material mass ratio of 1:8 (mol/mol). **Figure S4** (B) showed that the EE of HP-ICs reached the highest yield at 1 h. **Figure S4** (C) showed that the EE of HP-ICs reached the highest yield at 50 °C.

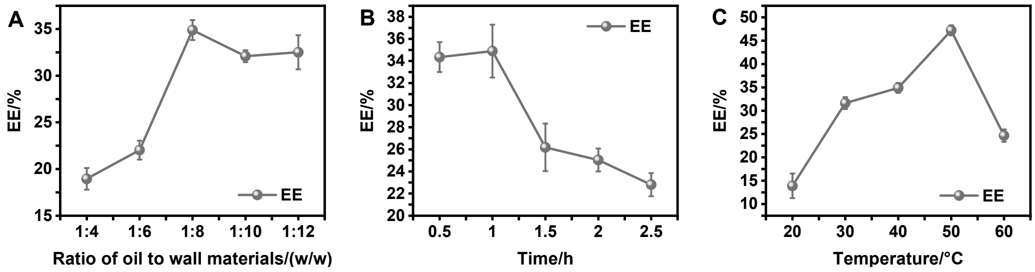

Figure S4. Effect of the (A) ratio of oil to wall materials, (B) time, (C) temperature on the EE of HP-ICs

As shown in **Table S4**, when the mass ratio of oil to wall materials, time, and temperature conditions were 1:10 (w/w), 1.5 h, and 30 °C, respectively, the EE reached the highest value.

Table S4. The design and results of the orthogonal formulation of HP-ICs [a]

Numbers	Factors				EE%
	A	B	C	D	
1	1（1:8）	1（0.5h）	1（30°C）	1	34.79 ± 1.32
2	1	2（1h）	2（40°C）	2	30.95 ± 3.25
3	1	3（1.5h）	3（50°C）	3	25.27 ± 1.98
4	2（1:10）	1	2	3	35.29 ± 1.36
5	2	2	3	1	38.95 ± 1.03
6	2	3	1	2	43.88 ± 2.49
7	3（1:12）	1	3	2	28.80 ± 3.67
8	3	2	1	3	34.40 ± 2.12
9	3	3	2	1	32.65 ± 1.44
k_1	30.34	32.96	37.69	35.46	
k_2	39.37	34.77	32.96	34.54	
k_3	31.95	33.93	31.01	31.65	
R	9.04	1.80	6.69	3.81	

[a] Note: A means the ratio of oil to wall materials, B means the time value, C means the temperature values, and D means blank column. k_1 is the mean values for level 1 of each factor; k_2 is the mean values for level 2 of each factor; k_3 is the mean values for level 3 of each factor. R is the difference between the maximum and minimum of the encapsulation efficiency (EE) for every level. R = max (k_i) − min (k_i).

S2.5. DM-ICs

Figure S5 (A) showed that the EE of DM-ICs reached the highest yield at the oil/wall material mass ratio of 1:12 (w/w). **Figure S5** (B) showed that the EE of DM-ICs reached the highest yield at 2 h. **Figure S5** (C) showed that the EE of DM-ICs reached the highest yield at 50 °C.

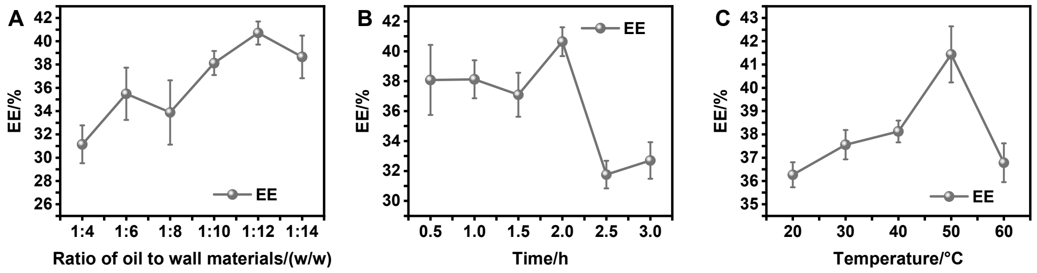

Figure S5. Effect of the (A) ratio of oil to wall materials, (B) time, (C) temperature on the EE of DM-ICs

As shown in **Table S5**, when the mass ratio of oil to wall materials, time, and temperature conditions were 1:12 (w/w), 1 h, and 50 °C, respectively, the EE of DM-ICs reached the highest value.

Table S5. The design and results of the orthogonal formulation of DM-ICs [a]

Numbers	Factors				EE%
	A	B	C	D	
1	1（1:10）	1（0.5h）	1（30℃）	1	42.73 ± 1.17
2	1	2（1h）	2（40℃）	2	21.37 ± 2.35
3	1	3（2h）	3（50℃）	3	34.14 ± 1.38
4	2（1:12）	1	2	3	45.42 ± 1.86
5	2	2	3	1	49.90 ± 0.85
6	2	3	1	2	41.54 ± 2.46
7	3（1:14）	1	3	2	26.95 ± 3.07
8	3	2	1	3	39.99 ± 1.12
9	3	3	2	1	27.16 ± 0.74
k_1	32.75	38.37	41.42	39.93	
k_2	45.62	37.08	31.32	29.95	
k_3	31.37	34.28	37.00	39.85	
R	14.25	4.09	10.10	9.97	

[a] Note: A means the ratio of oil to wall materials, B means the time value, C means the temperature values, and D means blank column. k_1 is the mean values for level 1 of each factor; k_2 is the mean values for level 2 of each factor; k_3 is the mean values for level 3 of each factor. R is the difference between the maximum and minimum of the encapsulation efficiency (EE) for every level. R = max (k_i) − min (k_i).
